# Arterial Stiffness and Anti-NR2/GluN2 Autoantibodies to N-Methyl-D-Aspartate Receptors as Markers of Vascular Aging and Cognitive Impairment in Arterial Hypertension

**DOI:** 10.3390/life16091418

**Published:** 2026-08-26

**Authors:** Tatiana Safronova, Sofia Mukhanova, Anna Bragina, Aida Tarzimanova, Alexander Ivannikov, Tatiana Vargina, Anna Pokrovskaya, Julia Rodionova, Natalia Druzhinina, Lyubov Vasilyeva, Alexander Cherepanov, Nikolay Dorokhov, Natalia Gureva, Natalia A. Dragomiretskaya, Irakli Zh. Loriya, Valery Podzolkov

**Affiliations:** 1 Department of Faculty Therapy No. 2, Sechenov First Moscow State Medical University (Sechenov University), 119048 Moscow, Russia; muhanova16@yandex.ru (S.M.); bragina_a_e@staff.sechenov.ru (A.B.); tarzimanova_a_i@staff.sechenov.ru (A.T.); vargina_t_s@staff.sechenov.ru (T.V.); pokrovskaya_a_e@staff.sechenov.ru (A.P.); rodionova_yu_n@staff.sechenov.ru (J.R.); druzhinina_n_a@staff.sechenov.ru (N.D.); vasileva_l_v_2@staff.sechenov.ru (L.V.); cherepanov_a_g@staff.sechenov.ru (A.C.); dorohovnikolay04@mail.ru (N.D.); gureva.2005@internet.ru (N.G.); dragomiretskaya_n_a@staff.sechenov.ru (N.A.D.); loriya_i_zh@staff.sechenov.ru (I.Z.L.); podzolkov_v_i@staff.sechenov.ru (V.P.); 2 N.V. Sklifosovsky Research Institute for Emergency Medicine, 129090 Moscow, Russia; ivannikov_a95@mail.ru

**Keywords:** arterial hypertension, CAVI, NR2 antibodies, MMSE scale, MoCA scale

## Abstract

Background: Arterial hypertension (HTN) is associated with accelerated vascular aging, cerebral microvascular injury, and cognitive decline. Anti-NR2/GluN2 (NR2) antibodies may be related to N-methyl-D-aspartate receptor (NMDAR)-mediated neuronal-ischemic injury, although their clinical significance in HTN requires further clarification. Objective: The aim of this study was to evaluate cross-sectional associations between arterial stiffness assessed by the cardio-ankle vascular index (CAVI), serum NR2 antibody levels, and cognitive performance assessed by the Montreal Cognitive Assessment (MoCA) and Mini-Mental State Examination (MMSE) in patients with essential HTN and controls. Methods: This single-center study included 175 adults aged 40–70 years: 128 patients with essential HTN and 47 controls. The median age was 58 [54; 66] years in the HTN group and 55 [47; 62] years in controls (*p* = 0.06). Men accounted for 52 patients (40.6%) in the HTN group and 14 participants (30.0%) in the control group (*p* = 0.22). Arterial stiffness was assessed using CAVI. Serum NR2 antibody levels were measured by enzyme-linked immunosorbent assay. Cognitive status was evaluated using MoCA and MMSE. Between-group comparisons, correlation analysis, and exploratory classification modeling were performed. Results: Patients with HTN had higher CAVI values than controls (8.3 [7.3; 9.4] vs. 7.3 [6.9; 7.9]; *p* = 0.001). Cognitive impairment was more frequent in hypertensive patients by both MoCA (52.3% vs. 27.7%; *p* = 0.003) and MMSE (48.7% vs. 28.3%; *p* = 0.01). NR2 antibody levels were higher in HTN (1.6 [1.15; 2.62] vs. 1.33 [1.02; 1.84] ng/mL; *p* = 0.03). CAVI inversely correlated with MoCA and MMSE scores. Conclusions: CAVI was higher in patients with HTN and showed modest inverse associations with cognitive scores. NR2 antibody levels were also higher in HTN; however, their relationship with cognitive impairment should be interpreted as exploratory and hypothesis-generating. Further longitudinal studies with neuroimaging are required to determine the clinical and prognostic significance of these findings.

## 1. Introduction

Arterial hypertension (HTN) is one of the leading modifiable factors of cardiovascular and cerebrovascular risk and is consistently associated with subsequent decline in cognitive function [1,2,3]. HTN-related brain injury develops through interrelated mechanisms, including endothelial dysfunction, small-vessel remodeling, impaired neurovascular coupling, chronic hypoperfusion, oxidative stress, and increased blood–brain barrier permeability [3,4,5].

Increased arterial stiffness is an important manifestation of vascular aging. By reducing the buffering capacity of large arteries and increasing pulsatile transmission to low-resistance organs, arterial stiffness may contribute to cerebral microvascular injury and cognitive decline [6,7,8]. The cardio-ankle vascular index (CAVI) is a non-invasive marker of arterial stiffness that reflects the condition of the arterial tree from the aorta to the lower-limb arteries and is considered relatively less dependent on blood pressure at the time of measurement [9,10,11]. Previous studies have also linked higher CAVI values with poorer executive function and mild cognitive decline [12,13,14].

Along with macrovascular parameters, markers potentially related to neuronal-ischemic injury are of interest. NR2 antibodies are directed against NR2/GluN2 subunits of NMDA receptors, which are involved in synaptic transmission, neuroplasticity, learning, and memory [15,16]. Glutamate excitotoxicity is also implicated in ischemic neuronal injury [17]. Previous studies have reported associations of NR2-related markers with transient ischemic attack, ischemic stroke, subclinical brain injury in HTN, and cerebral small vessel disease [18,19,20,21,22,23,24,25]. However, in the absence of neuroimaging, interpretation of NR2 antibodies as markers of structural brain damage should remain cautious.

Cognitive screening scales such as MoCA and MMSE remain essential tools for detecting cognitive impairment, although their sensitivity depends on the stage and profile of cognitive deficit [26,27,28,29,30]. Therefore, combined assessment of vascular stiffness, NR2 antibodies, and cognitive testing may help characterize cardio-cerebral associations in HTN.

The aim of this study was to evaluate cross-sectional associations between arterial stiffness assessed by CAVI, serum NR2 antibody levels, and cognitive performance assessed by MoCA and MMSE in patients with essential HTN and controls.

## 2. Materials and Methods

The study was conducted at the Clinical Center of I.M. Sechenov First Moscow State Medical University of the Ministry of Health of Russia (Sechenov University), University Clinical Hospital No. 4, Clinic of Faculty Therapy No. 2, Department of Faculty Therapy No. 2, N.V. Sklifosovsky Institute of Clinical Medicine.

The study was performed in accordance with Good Clinical Practice standards and in compliance with the ethical principles delineated in the Declaration of Helsinki. The research protocol was approved by the Local Ethics Committee of the Federal State Autonomous Educational Institution of Higher Education “I.M. Sechenov First Moscow State Medical University” of the Ministry of Health of Russia (Sechenov University), protocol No. 01-25, dated 23 January 2025.

The inclusion criteria were age over 18 years, signed informed consent to participate in the study, and, for the study group, essential HTN. Non-inclusion/exclusion criteria included secondary HTN, chronic heart failure, coronary artery disease, atrial fibrillation and life-threatening arrhythmias, chronic kidney disease stage 3 or higher, diabetes mellitus or impaired glucose tolerance, left ventricular ejection fraction below 50%, psychiatric disorders, severe somatic, neurological, or oncological diseases, severe hearing or visual impairment, and lack of patient consent. The presence of brachiocephalic artery stenosis greater than 50% was also an exclusion criterion.

Participants were recruited in an outpatient setting. The study included patients with controlled HTN and apparently healthy individuals undergoing routine preventive medical examinations. These individuals formed the control group. Initially, 100 individuals who self-identified as healthy were screened for inclusion in the control group. However, 53 were excluded after screening because newly detected conditions were identified, including diabetes mellitus or impaired glucose tolerance, atrial fibrillation, left ventricular ejection fraction below 50%, and cardiac rhythm disturbances. The same inclusion and exclusion criteria were applied in both groups.

A total of 175 participants aged 40 to 70 years were included in this single-center study: 128 patients with essential HTN constituted the first group, and 47 participants without HTN constituted the control group. All patients underwent examination in accordance with clinical guidelines for HTN in adults [1]. Patients with HTN were adherent to pharmacological therapy and had been receiving treatment for at least one year. All patients in the study group received antihypertensive drugs, including ACE inhibitors or angiotensin receptor blockers, as well as thiazide diuretics; approximately 70% of patients also received calcium channel blockers. At study inclusion, target blood pressure values had been achieved in these patients.

Arterial stiffness was assessed using the cardio-ankle vascular index (CAVI), which was determined with the VaSera VS-1500 (Fukuda Denshi Co., Ltd., Tokyo, Japan) [9,10,11]. Estimated vascular age was also obtained using the device algorithm and was treated as a descriptive parameter rather than a clinical endpoint.

Serum NR2 antibody levels were measured by enzyme-linked immunosorbent assay (NR2Ab-ELISA kit, DRD LLC, Moscow, Russia) according to the manufacturer’s instructions and previously published recommendations [18,21,22]. For analysis, patients were divided into two groups according to NR2 antibody levels using a threshold of 1.8 ng/mL: low NR2 antibody levels (<1.8 ng/mL) and high NR2 antibody levels (≥1.8 ng/mL) [18,19,21,22].

Cognitive status was assessed using the Montreal Cognitive Assessment (MoCA) and the Mini-Mental State Examination (MMSE) [26,27]. Validated Russian-language versions of MoCA and MMSE were used. The investigator administering MoCA had completed the MoCA Cognition training course, authorizing the administration and interpretation of MoCA test results. All participants had secondary or higher education; therefore, no additional correction for low educational level was applied. Age-specific correction was not applied because the study included participants younger than 70 years. The investigators who performed cognitive testing were blinded to CAVI and NR2 antibody results.

The maximum score for both scales is 30, corresponding to optimal cognitive function. For MoCA, scores of 18–25 indicated mild cognitive impairment, 10–17 indicated moderate cognitive impairment, and scores below 10 indicated severe cognitive impairment; MoCA scores ≥ 26 were considered normal, whereas MoCA scores ≤ 25 were interpreted as the presence of cognitive impairment [27,28,30]. For MMSE, scores of 24–27 were classified as mild cognitive impairment, 20–23 as moderate cognitive impairment, 11–19 as severe cognitive impairment, and scores below 10 as very severe impairment; MMSE scores ≥ 28 were considered normal, whereas MMSE scores ≤ 27 were interpreted as the presence of cognitive impairment [26,29].

A general questionnaire was administered to all participants and included questions regarding current or previous stress and depression. According to the responses provided, none of the participants reported depression. Sleep apnea was also assessed using the general questionnaire, which included questions about both a previously established diagnosis and symptoms such as snoring, witnessed episodes of breathing cessation, or choking during sleep; however, this information was based on patient self-report and medical history.

Statistical analysis was performed using SPSS version 22. Quantitative variables were assessed for normality using the Shapiro–Wilk test. Variables that did not follow a normal distribution were described using the median (Me) and the first and third quartiles [Q1; Q3]. Categorical variables are presented as absolute and relative (%) frequencies. The Mann–Whitney U test was used for between-group comparisons. Nominal data were analyzed using Pearson’s chi-square test and Fisher’s exact test. Correlation analysis was performed using Spearman’s rank correlation coefficient for quantitative variables and the point-biserial Pearson correlation coefficient for binary variables. We used the Bonferroni correction to control the family-wise error rate because multiple correlation analyses were performed. Since 45 pairwise comparisons were carried out, the adjusted significance threshold after Bonferroni correction was set at α_adj (Bonferroni) = 0.0011 (0.05/45). Correlations with Bonferroni-adjusted *p*-values ≤ 0.05 were considered statistically significant. No formal multiplicity correction was applied to between-group comparisons, which were considered descriptive and were used to characterize baseline demographic, clinical, laboratory, and instrumental differences between the HTN and control groups. Initially, candidate predictors were ranked according to their importance using a Random Forest algorithm. Feature importance scores were used to identify the variables with the greatest contribution to outcome prediction. The highest-ranked predictors were subsequently selected for inclusion in the final model. A logistic regression classifier was then developed using the selected variables. Continuous predictors were standardized using the StandardScaler procedure before model fitting. The final model included NR2 concentration, indexed left atrial volume, the presence of arterial hypertension, C-reactive protein level, and albumin concentration. Model performance was assessed in terms of discrimination using the area under the receiver operating characteristic curve. The critical level of statistical significance was set at *p* ≤ 0.05. Analyses were conducted using available complete-case data for each variable. To evaluate multicollinearity among the explanatory variables in the regression model, the variance inflation factor (VIF) and tolerance were computed for each independent predictor. Observations with missing values were excluded from the corresponding analysis, and no imputation was performed. The classification model included only participants for whom serum NR2 antibody measurements and all model variables were available.

## 3. Results

The study included 175 participants: 128 patients with HTN and 47 controls. The groups were comparable in age: the median age was 58 [54; 66] years in the HTN group and 55 [47; 62] years in the control group (*p* = 0.06) Table 1. No significant differences were observed in sex distribution or smoking status: men accounted for 52 patients (40.6%) and 14 participants (30.0%), respectively (*p* = 0.22), and smokers for 42 (33.6%) and 14 (29.8%) participants, respectively (*p* = 0.63). Among patients with HTN, the median disease duration was 9 [4; 15] years; grade 1 HTN was diagnosed in 9 (7%) patients, grade 2 in 38 (30%), and grade 3 in 81 (63%). Body mass index was significantly higher in patients with HTN than in controls: 29.4 [26; 32.8] vs. 24.7 [22.5; 29.5] kg/m^2^ (*p* = 0.001).

Analysis of structural and functional cardiac parameters revealed more pronounced myocardial and left-heart remodeling in patients with HTN. Interventricular septal thickness was 0.9 [0.8; 1] cm versus 0.8 [0.8; 0.8] cm in the control group (*p* = 0.001), and left ventricular posterior wall thickness was 0.8 [0.8; 1] versus 0.8 [0.8; 0.8] cm (*p* = 0.025). Left ventricular ejection fraction remained within preserved values in both groups, but was statistically lower in patients with HTN: 63 [62; 65]% versus 65 [64; 65]% (*p* = 0.025). Left atrial end-systolic volume and left atrial end-systolic dimension were also higher in the HTN group: 47.5 [43; 54] mL versus 43 [34; 45] mL (*p* = 0.001) and 3.4 [3.2; 3.7] cm versus 3.2 [3.1; 3.4] cm (*p* = 0.002), respectively. No differences were found in left ventricular mass index.

Lipid and carbohydrate metabolism parameters did not differ significantly between the groups. At the same time, patients with HTN had higher urea levels: 5.4 [4.4; 6.3] mmol/L versus 4.7 [3.2; 5.6] mmol/L in controls (*p* = 0.007) and a lower estimated glomerular filtration rate: 75.6 [64.9; 86.3] versus 84 [70.5; 94.4] mL/min/1.73 m^2^ (*p* = 0.006). Glucose, total cholesterol, low-density lipoprotein cholesterol, creatinine, C-reactive protein, and total protein levels did not differ significantly.

Arterial stiffness assessed by CAVI was higher in patients with HTN. The median CAVI was 8.3 [7.3; 9.4] in the HTN group and 7.3 [6.9; 7.9] in the control group (*p* = 0.001). In categorical analysis, normal CAVI values < 8 were recorded in 49 (43.4%) patients with HTN and in 33 (76.7%) controls. Intermediate CAVI values of 8.0–8.9 were observed in 28 (24.8%) and 8 (18.6%) participants, respectively. Markedly elevated CAVI values ≥ 9 were observed predominantly in patients with HTN: 36 (31.9%) versus 2 (4.7%) in the control group (*p* = 0.001). Estimated vascular age was treated as a descriptive parameter and was not considered a clinical endpoint.

Cognitive status was assessed using the MoCA and MMSE scales. In the overall sample, the frequency of cognitive impairment depended on the diagnostic instrument used: cognitive impairment was detected in 45.7% of participants by MoCA and in 42.9% by MMSE. Patients with HTN had cognitive impairment significantly more frequently than controls: 52.3% versus 27.7% by MoCA (*p* = 0.003) and 48.7% versus 28.3% by MMSE (*p* = 0.01).

NR2 antibody levels were higher in patients with HTN than in controls: 1.6 [1.15; 2.62] versus 1.33 [1.02; 1.84] ng/mL (*p* = 0.03). Using the threshold NR2 antibody level of ≥1.8 ng/mL, elevated values were detected in 24 (47%) patients with HTN and in 9 (31%) controls; NR2 antibody levels < 1.8 ng/mL were observed in 27 (53%) and 20 (69%) participants, respectively. When NR2 antibody levels were compared with cognitive screening results, some patients with elevated NR2 antibodies did not have cognitive impairment according to MoCA or MMSE. This finding should be interpreted as hypothesis-generating only, because the study did not include brain MRI, cerebral small vessel disease assessment, or detailed neuropsychological testing.

Correlation analysis (Table 2) showed that higher CAVI was associated with lower cognitive scores. In the overall sample, CAVI correlated negatively with MoCA (r = −0.300; *p* = 0.001) and MMSE (r = −0.200; *p* = 0.016) scores. In patients with HTN, the association between CAVI and MoCA was more pronounced (r = −0.400; *p* = 0.001). Similar negative correlations were obtained for MMSE among patients with HTN (r = −0.220; *p* = 0.025).

CAVI demonstrated positive correlations with markers of cardiac remodeling: left ventricular mass index (r = 0.402; *p* < 0.01), interventricular septal thickness (r = 0.322; *p* < 0.01), left ventricular posterior wall thickness (r = 0.379; *p* < 0.01), left atrial end-systolic dimension (r = 0.330; *p* < 0.01), and left atrial volume (r = 0.426; *p* < 0.01). A negative association between CAVI and glomerular filtration rate was also identified (r = −0.215; *p* < 0.05). These findings confirm the relationship between arterial stiffness and signs of cardiovascular remodeling and target-organ damage.

Cognitive status parameters also correlated with echocardiographic measures. MMSE showed negative associations with left ventricular mass index (r = −0.322; *p* < 0.01), interventricular septal thickness (r = −0.283; *p* < 0.01), left ventricular posterior wall thickness (r = −0.306; *p* < 0.01), and left atrial volume (r = −0.240; *p* < 0.05). MoCA scores correlated negatively with left atrial end-systolic dimension (r = −0.286; *p* < 0.01), left atrial volume (r = −0.226; *p* < 0.05), and urea level (r = −0.211; *p* < 0.05).

After applying the Bonferroni correction for multiple comparisons (adjusted threshold α = 0.0011), all correlations with initial *p*-values of 0.001 remained statistically significant after correction, indicating the robustness of the strongest observed associations, particularly those involving CAVI and cardiovascular parameters.

To evaluate the association with prevalent cognitive impairment, defined as a MoCA score below 26 points at baseline, an exploratory classification model based on binary logistic regression was developed. The presence of cognitive impairment, coded as a binary outcome, was used as the target variable. This model was not designed to predict future cognitive decline. The model included NR2 concentration, indexed left atrial volume, the presence of HTN, C-reactive protein level, and albumin concentration. NR2 concentration and the presence of HTN were included a priori in accordance with the study aim, whereas the remaining clinical, laboratory, and instrumental parameters were used to improve classification performance.

**Figure 1:**
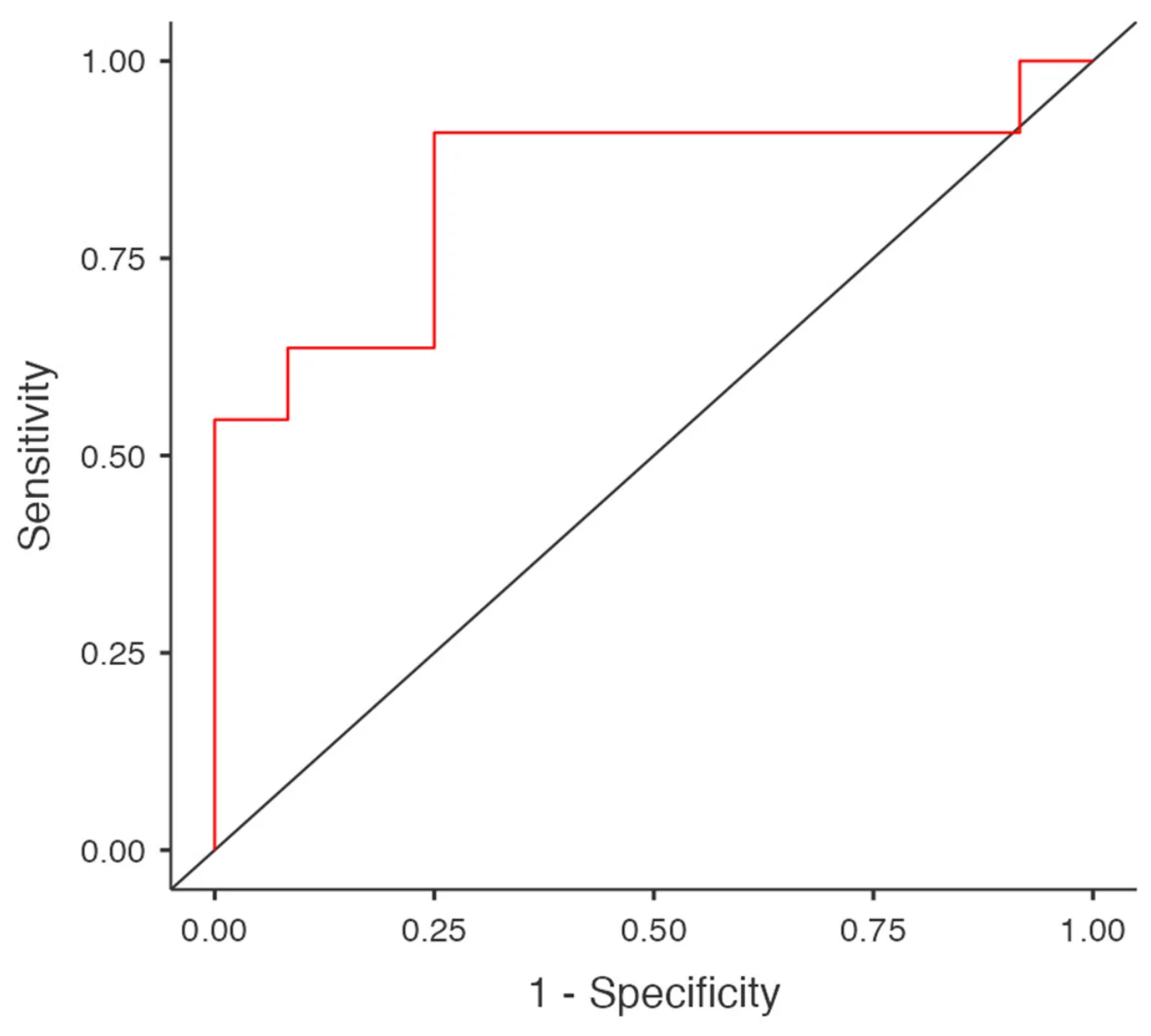
Area under the curve of the developed multivariable logistic regression model.

**Figure 2:**
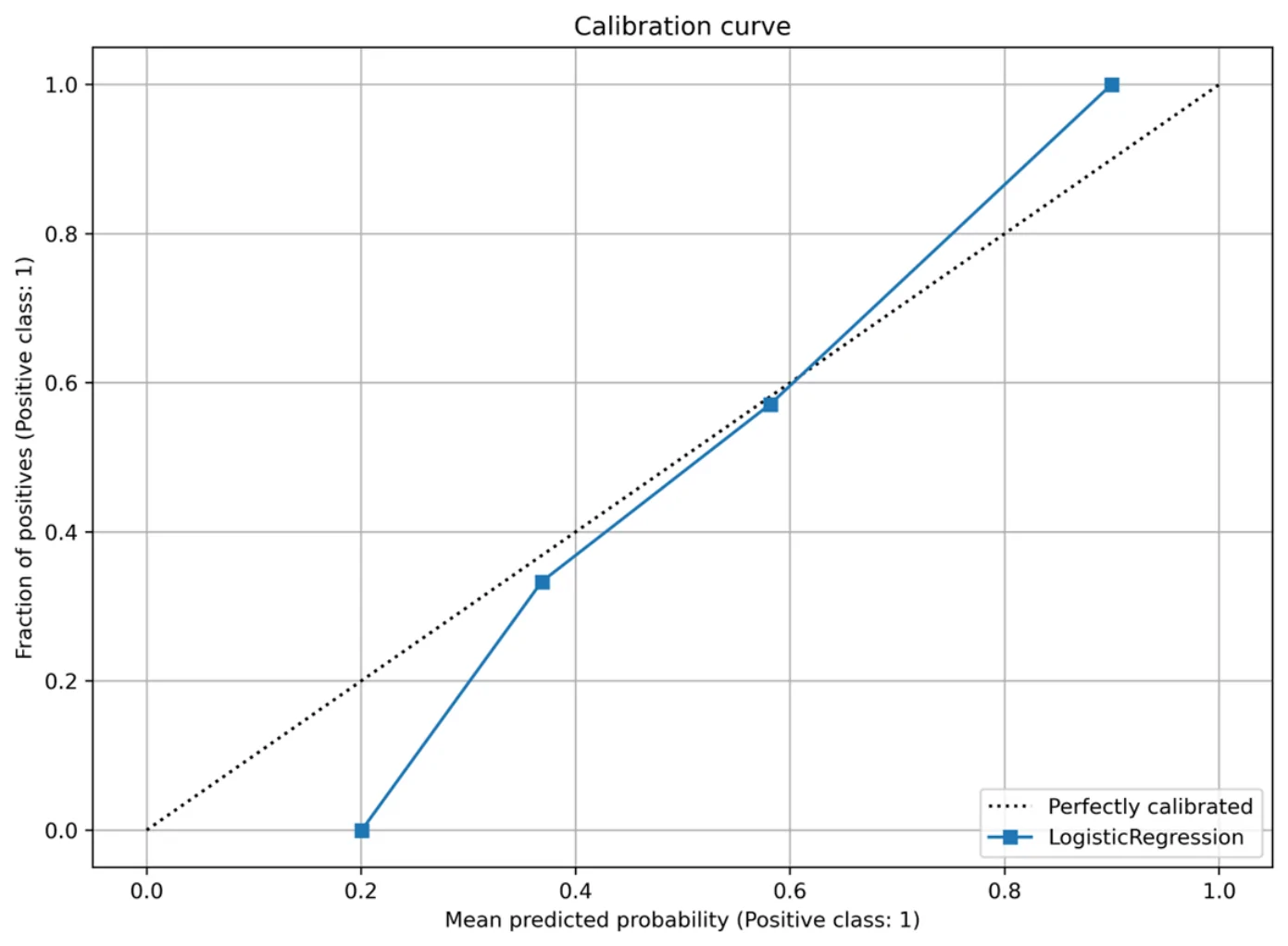
Calibration plot of the exploratory logistic regression classification model. The dashed diagonal line represents perfect agreement between predicted and observed event probabilities. The solid line represents the observed calibration of the developed logistic regression model.

The exploratory classification model demonstrated good discriminatory ability in distinguishing participants with and without prevalent cognitive impairment. The area under the ROC curve was 0.841 (Figure 1). At a predicted probability threshold of 0.36, the model sensitivity reached 72.7%, specificity 75.0%, and overall classification accuracy 73.9%. McFadden’s pseudo-R2 was 0.281, model deviance was 22.9, and the Akaike information criterion was 34.9. Calibration analysis demonstrated satisfactory agreement between predicted and observed probabilities of cognitive impairment (Figure 2). The Brier score was 0.181, indicating acceptable overall accuracy of probabilistic predictions.

All predictors demonstrated low VIF values (1.12–1.29) and high tolerance values (0.774–0.892), indicating the absence of significant multicollinearity among the explanatory variables (Table 3).

## 4. Discussion

The present cross-sectional results demonstrate that patients with HTN, despite achieving target blood pressure values on combined antihypertensive therapy, had higher CAVI values, more pronounced cardiac remodeling, and a higher prevalence of cognitive impairment according to MoCA and MMSE. These findings are consistent with the concept that HTN-related cardio-cerebral alterations may develop over a prolonged period and may not be fully reflected by office blood pressure control alone [1,2,3,5].

All patients with HTN were adherent to antihypertensive therapy and had been receiving treatment for at least one year. The treatment regimen included ACE inhibitors or angiotensin receptor blockers and thiazide diuretics in all patients, while approximately 70% also received calcium channel blockers. Since the study did not include an untreated HTN group and did not involve longitudinal follow-up, the independent effects of antihypertensive or nephroprotective therapy on CAVI, renal function, inflammation, and cognitive performance could not be assessed.

Higher CAVI values in patients with HTN indicate increased arterial stiffness as one phenotype of hypertensive vascular remodeling. The observed associations between CAVI and cognitive scores were modest and should not be interpreted as evidence of diagnostic utility. Rather, these findings support further investigation of arterial stiffness as one of several vascular factors associated with cognitive performance in HTN [8,12,13,14].

Higher NR2 antibody levels in patients with HTN may reflect processes related to neurovascular injury; however, in the absence of brain MRI, cerebral small vessel disease assessment, and detailed neuropsychological testing, this interpretation remains hypothesis-generating. The dissociation between elevated NR2 antibody levels and normal MoCA/MMSE scores in some participants should therefore not be interpreted as definitive evidence of preclinical cerebral damage. Further studies are required to clarify the clinical significance of this finding.

The exploratory classification model demonstrated an association between NR2 antibodies and prevalent cognitive impairment at baseline rather than the prediction of future cognitive decline. Although the model showed acceptable discrimination and calibration, it requires prospective validation in larger cohorts before any diagnostic or prognostic interpretation can be made.

### Study Limitations

This study has several limitations. It was a single-center cross-sectional study; therefore, causality, temporality, and prediction of future cognitive decline cannot be established. No longitudinal follow-up was performed. The control group was smaller than initially planned, and residual selection bias cannot be excluded. The study did not include brain MRI, assessment of cerebral small vessel disease, or a detailed neuropsychological battery. All patients with HTN were treated, and there was no untreated HTN group; consequently, the independent influence of antihypertensive therapy could not be assessed. The classification model should therefore be regarded as exploratory and requires external validation.

## 5. Conclusions

1. Patients with HTN had higher CAVI values and a higher frequency of cognitive impairment according to MoCA and MMSE compared with controls.
2. CAVI showed modest inverse associations with cognitive scores, with the strongest association observed between CAVI and MoCA in patients with HTN.
3. NR2 antibody levels were higher in patients with HTN; however, their interpretation as markers related to neurovascular injury remains exploratory.
4. Elevated NR2 antibody levels in the presence of normal MoCA or MMSE scores should be considered a hypothesis-generating observation rather than evidence of preclinical cerebral damage.
5. Prospective studies with brain MRI, assessment of cerebral small vessel disease, and detailed neuropsychological testing are required to determine the clinical and prognostic significance of CAVI and NR2 antibodies in HTN.

## Figures and Tables

**Table 1:** Baseline demographic, clinical, laboratory, and instrumental characteristics of the study groups.

Parameters, Me [Q1; Q3]/*n* (%)	HTN Group (*n* = 128)	Control Group (*n* = 47)	*p*-Value
Age, years	58 [54; 66]	55 [47; 62]	0.06
Men	52 (40.6%)	14 (30.0%)	0.22
HTN grade 1/2/3	9 (7%)/38 (30%)/81 (63%)	-	-
Duration of HTN, years	9 [4; 15]	-	-
BMI, kg/m^2^	29.4 [26; 32.8]	24.7 [22.5; 29.5]	0.001 *
Smoking	42 (33.6%)	14 (29.8%)	0.63
Glucose, mmol/L	5.3 [4.9; 5.8]	5.1 [4.6; 5.7]	0.06
Total cholesterol, mmol/L	5.4 [4.6; 6.2]	5.3 [4.5; 6.1]	0.7
LDL-C, mmol/L	3.2 [2.7; 4.2]	3.4 [3; 3.6]	0.76
Urea, mmol/L	5.4 [4.4; 6.3]	4.7 [3.2; 5.6]	0.007 *
Creatinine, umol/L	82 [73; 92.3]	78.5 [68.8; 84]	0.08
eGFR, mL/min/1.73 m^2^	75.6 [64.9; 86.3]	84 [70.5; 94.4]	0.006 *
Total protein, g/L	72 [66; 75]	69 [67; 74]	0.45
CRP, mg/L	2.8 [1; 4]	1.5 [0.8; 6.5]	0.4
LVMI, g/m^2^	71.8 [61.8; 87.6]	67.8 [60.5; 71]	0.22
IVS thickness, cm	0.9 [0.8; 1]	0.8 [0.8; 0.8]	0.001 *
LVPW thickness, cm	0.8 [0.8; 1]	0.8 [0.8; 0.8]	0.025 *
LVEF, %	63 [62; 65]	65 [64; 65]	0.025 *
LA end-systolic volume, mL	47.5 [43; 54]	43 [34; 45]	0.001 *
LA end-systolic dimension, cm	3.4 [3.2; 3.7]	3.2 [3.1; 3.4]	0.002 *
NR2 antibodies, ng/mL	1.6 [1.15; 2.62]	1.33 [1.02; 1.84]	0.03
CAVI	8.3 [7.3; 9.4]	7.3 [6.9; 7.9]	0.001 *
SBP, mmHg	134 [126; 142]	126 [120; 134]	0.001 *
DBP, mmHg	87 [80; 93]	81 [76; 86]	0.001 *
MMSE score	28 [26.5; 29]	28.5 [27; 29]	0.012 *
MoCA score	25 [24; 27]	27 [25; 28]	0.003 *

Note: * *p*-value < 0.05. BMI, body mass index; LDL-C, low-density lipoprotein cholesterol; eGFR, estimated glomerular filtration rate; CRP, C-reactive protein; LVMI, left ventricular mass index; IVS, interventricular septum; LVPW, left ventricular posterior wall; LVEF, left ventricular ejection fraction; LA, left atrial; SBP, systolic blood pressure; DBP, diastolic blood pressure.

**Table 2:** Correlation analysis of CAVI, cognitive scores, echocardiographic parameters, and renal/biochemical markers.

Correlation Pair	Sample	r	*p*-Value	p-adj (Bonferroni)
CAVI–MoCA	Overall sample	−0.300	0.001	0.045
CAVI–MMSE	Overall sample	−0.200	0.016	0.720
CAVI–MoCA	Patients with HTN	−0.400	0.001	0.045
CAVI–MMSE	Patients with HTN	−0.220	0.025	1.125
CAVI–LVMI	Overall sample	0.402	0.001	0.045
CAVI–IVS thickness	Overall sample	0.322	0.001	0.045
CAVI–LVPW thickness	Overall sample	0.379	0.001	0.045
CAVI–LA end-systolic dimension	Overall sample	0.330	0.001	0.045
CAVI–LA volume	Overall sample	0.426	0.001	0.045
CAVI–eGFR	Overall sample	−0.215	0.013	0.585
MMSE–LVMI	Overall sample	−0.322	0.001	0.045
MMSE–IVS thickness	Overall sample	−0.283	0.004	0.180
MMSE–LVPW thickness	Overall sample	−0.306	0.002	0.090
MMSE–LA volume	Overall sample	−0.240	0.017	0.765
MoCA–LA end-systolic dimension	Overall sample	−0.286	0.003	0.135
MoCA–LA volume	Overall sample	−0.226	0.022	0.990
MoCA–Urea	Overall sample	−0.211	0.012	0.540

Note: CAVI, cardio-ankle vascular index; HTN, hypertension; MoCA, Montreal Cognitive Assessment; MMSE, Mini-Mental State Examination; LVMI, left ventricular mass index; IVS, interventricular septum; LVPW, left ventricular posterior wall; LA, left atrial; eGFR, estimated glomerular filtration rate. p-adj, Bonferroni-adjusted *p*-value.

**Table 3:** Assessment of multicollinearity among predictors included in the exploratory logistic regression classification model.

Predictor	VIF	Tolerance
NR2 antibodies, ng/mL	1.13	0.882
Left atrial volume index, mL/m^2^	1.26	0.796
HTN, 0 = no, 1 = yes	1.29	0.774
C-reactive protein, mg/L	1.25	0.802
Albumin, g/L	1.12	0.892

Note: VIF, variance inflation factor; HTN, hypertension.

## Data Availability

The datasets used and/or analyzed during the current study are available from the corresponding author upon reasonable request.

## Abbreviations

The following abbreviations are used in this manuscript:

CAVI	cardio-ankle vascular index
MMSE	Mini-Mental State Examination
MoCA	Montreal Cognitive Assessment
AH/HTN	arterial hypertension/hypertension
